# 3D-Bioprinted Hydrogels Based on Calcium Alginate and Turmeric (*Curcuma longa* L.): Comprehensive Structural, Functional, and Biological Evaluation for Advanced Wound Healing Applications

**DOI:** 10.1021/acsomega.6c00044

**Published:** 2026-03-16

**Authors:** Rafaela Prediger dos Anjos, Paula de Abreu Fernandes, Hernane da Silva Barud, Marina de Lima Fontes, Marília Gonçalves Cattelan, Marcia Regina de Moura, Fauze Ahmad Aouada

**Affiliations:** † Group of Composites and Hybrid Nanocomposites (GCNH), 28108 Universidade Estadual Paulista (Unesp), Av. Brasil Sul, 56—Centro, Ilha Solteira, São Paulo 15385-007, Brazil; ‡ Laboratory of Biopolymers and Biomaterials (BioPolMat)—Uniara, R, University of Araraquara, Carlos Gomes, 1338—Centro, Araraquara, São Paulo 14801-340, Brazil; § Food Microbiology Laboratory, Universidade Estadual Paulista (Unesp), Rua Cristóvão Colombo, 2265—Jardim Nazareth, Sao Jose do Rio Preto, São Paulo 15054-000, Brazil

## Abstract

Sodium alginate polysaccharide is widely used for its ability to form hydrogels in the presence of calcium ions, showing excellent liquid retention properties. At the same time, *Curcuma longa* L. (CR) is known for its anti-inflammatory, antioxidant, and antimicrobial activities. Considering these characteristics, this study aimed to develop hydrogels from the combination of calcium alginate (CA) and CR through 3D bioprinting. The samples were characterized for their structural, spectroscopic, morphological, hydrophilic, thermal, and biological properties. UV–Vis spectroscopy monitored CR release, fitting to classical kinetic models. The release profile was concentration-dependent, where the 1.0% (w/v) CR hydrogel exhibited a faster and higher release while the 0.5% (w/v) CR formulation provided a slower and more controlled release. Kinetic modeling demonstrated that the Korsmeyer–Peppas model provided the best fit (*R* ^2^ > 0.99), with release exponents (*n* ≈ 0.50–0.54) consistent with Fickian to anomalous diffusion. The Higuchi model also correlated well (*R* ^2^ ≈ 0.86–0.89), reinforcing a diffusion-driven release mechanism. Biological assays revealed that 1.0% (w/v) CR hydrogels exhibited significant antibacterial activity against *Staphylococcus aureus*, with inhibition halos up to 12.67 mm but showed cytotoxicity to L929 fibroblasts (35% viability). In contrast, the 0.5% (w/v) CR hydrogel maintained biocompatibility (80% viability) while enabling sustained release of CR. Altogether, these findings highlight the versatility of CA/CR hydrogels, which combine swelling capacity, thermal stability, and controlled release of CR. Such systems are promising as advanced wound dressings, addressing the challenge of balancing antimicrobial efficacy with cellular biocompatibility. Future studies should include in vivo validation and testing under simulated chronic wound conditions to confirm their long-term safety and therapeutic potential.

## Graphical abstract

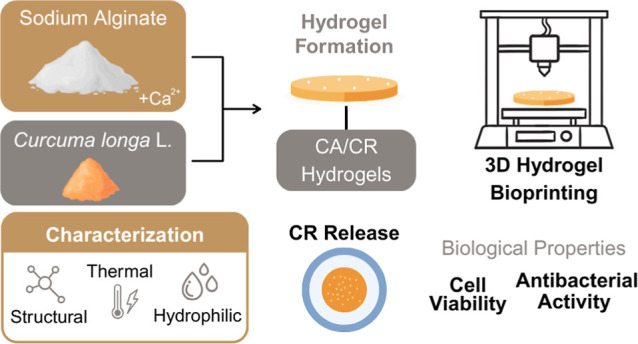

## 1 Introduction

Chronic wounds, particularly diabetic foot ulcers, represent a significant public health challenge due to their psychological and socioeconomic impact. These lesions, characterized by delayed healing, lead to reduced patient quality of life and increased healthcare costs, often associated with serious complications such as amputations.^1^ The healing process is complex, encompassing hemostasis, inflammation, proliferation, and remodeling phases. Each one is essential for tissue regeneration.^2–4^ Failure to transition from the inflammatory to the proliferative stage, often observed in chronic wounds, leads to persistent inflammation and poor tissue repair.

Modern dressings aim to maintain a moist environment, prevent infection, and support cellular processes, while traditional dressings such as gauze often adhere to wounds and offer limited protection. On the other hand, hydrogel- and alginate-based dressings provide superior biocompatibility, antimicrobial properties, and sustained drug release capabilities, significantly improving the wound healing process.^5–7^ Hydrogels, composed of hydrophilic polymer networks, can retain large amounts of water, offering a favorable microenvironment for cell proliferation and wound healing.^8,9^

Technological advancements, such as 3D printing, have enabled the fabrication of complex hydrogel structures for tissue regeneration.^10^ Natural polymers like cellulose, chitosan, and gelatin-based hydrogels are especially attractive due to their biodegradability and biofunctionality.^11,12^ Recent strategies include the incorporation of antimicrobial agents or bioactive molecules, such as zinc ions or natural antioxidants, into the hydrogel matrix to enhance antibacterial activity and reduce oxidative stress.^13,14^

Among the most promising materials, calcium alginate (CA) stands out for its unique three-dimensional “egg-box” structure, formed through ionic cross-linking with divalent cations such as Ca^2+^.^15^ CA hydrogels exhibit excellent moisture retention, hemostatic potential, and ability to modulate the inflammatory response, thereby accelerating granulation and epithelialization.^16^ In addition to wound healing, CA is extensively investigated in drug delivery systems, regenerative medicine, and protein encapsulation.^17,18^

*Curcuma longa* L. (CR), a medicinal plant widely known for its anti-inflammatory, antimicrobial, and antioxidant properties, has gained attention in recent wound care research. The primary bioactive compound of CR is the curcumin. This molecule displays potent free radical scavenging ability, promoting collagen deposition and accelerating tissue regeneration.^19^ However, curcumin’s poor aqueous solubility and photodegradation in alkaline pH challenge its direct biomedical application.^20^ Strategies to stabilize curcumin include its incorporation into polymeric systems, improving bioavailability and preserving its therapeutic efficacy^21,22^

Experimental studies have demonstrated that curcumin-loaded biomaterials reduce inflammation and oxidative stress, enhance angiogenesis, and exhibit significant antibacterial activity, including against methicillin-resistant *Staphylococcus aureus* (MRSA).^23,24^ In diabetic animal models, curcuminoids promoted rapid wound closure, increased granulation tissue formation, and improved collagen organization.^19,21^

The integration of CA and CR into hydrogel matrices represents a promising strategy for the development of multifunctional dressings. With the support of 3D bioprinting, these systems can be tailored to match the wound morphology and deliver therapeutics with spatial precision. This technology enables the fabrication of patient-specific constructs with complex geometries and bioactive components, mimicking the native extracellular matrix and enhancing tissue regeneration.^25,26^

3D-printed dressings are advantageous due to their customization and personalization ability to respond to different sizes, formats, and topographies of wounds, having great benefit for complex wounds in challenging areas. The ability to control the architecture and porosity of the dressing influences the ideal microenvironment for wound healing, promoting gas exchange and regulating the moisture of the wound.^27^ There is a cost-benefit in the use of 3D printing that brings the additional benefit of reducing material waste and production costs.^28^ Overall, CA- and CR-based bioengineering hydrogels manufactured by 3D printing provide a sustainable and effective platform for the next generation of wound healing therapies.

Although alginate-based hydrogel scaffolds are already consolidated in the literature due to their biocompatibility and network formation capacity, the main novelty of this study lies in the strategic incorporation of turmeric as a bioactive to functionalize this matrix. This approach aims to overcome the critical limitations of curcumin, such as its low water solubility and high susceptibility to chemical degradation, ensuring greater stability and allowing a controlled–sustained release.^29^ Although studies on complex matrices^30^ have shown that curcumin enhances cell proliferation, collagen synthesis, and antibacterial activity of the multifunctional hydrogel containing carboxymethylcellulose and polycaprolactone, this proposal is distinguished by its integration of curcumin into the alginate system. Such a combination offers the advantage of combining the structural and mechanical benefits of alginate with the multifunctional therapeutic properties of turmeric in a precisely controlled 3D architecture,^31^ resulting in a material superior to conventional hydrogels for biomedical applications.

The goal of this study was to develop different *scaffolds* based on 3D-bioprinted hydrogels based on calcium alginate and *Curcuma longa* L. In order to achieve this objective, the spectroscopic and structural properties of the matrices were investigated through Fourier-transform infrared spectroscopy (FTIR) and X-ray diffraction (XRD). The effect of CR on thermal stability was analyzed by thermogravimetric (TG) and differential thermogravimetric (DTG) analysis. The hydrophilic properties were assessed by determining the swelling degree in water (swelling cycles) and under different pH ranges (2, 4, 6, 8, and 10). The release of CR in solution was evaluated using UV–visible spectroscopy, while the morphology of the scaffolds was examined by scanning electron microscopy (SEM), and the cytocompatibility was assessed through a cell viability assay using an indirect method. The findings obtained contribute to current insights regarding the CA/CR 3D hydrogel and its applications in the medical field.

## 2 Materials and Methods

### 2.1 Materials

Sodium alginate (SA) (Mv: 85 000 g/mol) was obtained from Cromoline Química Fina-Brazil. The turmeric dried extract used was purchased from a local pharmacy. Anhydrous calcium chloride was purchased from Vetec (Brazil). Potassium bromide (KBr) was obtained from Sigma-Aldrich. Absolute ethyl alcohol P.A. was purchased from Exodus Cientifica Qumica Fina (Brazil). Chloramphenicol was obtained from the company Laborcin. Agar Müeller-Hinton, was obtained from HiMedia Laboratories. The reagents were used as received without any purification.

### 2.2 Ink Manufacturing Hydrogels

Initially, the CR was dispersed in an ethanol/water solution in the following proportions: 1:9, 2:8, 3:7, 6:4, 7:3, and 9:1 (% v/v). None of these dispersions were stable; that is, CR precipitation was observed in all of the proportions mentioned.

To ensure stability and prevent precipitation during the formation of the hydrogel, a viscous solution containing the compounds was used. To prepare these solutions, sodium alginate was dissolved in deionized water under constant agitation between 1500 and 2000 rpm. Then, the CR was added slowly in the solution containing half the total amount of SA, maintaining continuous agitation to ensure uniform dispersion of the particles. In this solution, the rest of the SA was added, and agitation was maintained until complete solubilization. The high viscosity provided by SA, associated with the electrostatic interactions and hydrogen bonds between them, kept the CR particles dispersed, preventing the formation of precipitates and favoring the uniform formation of the hydrogel.

Five different concentrations were prepared for the hydrogel solution (0.1, 0.25, 0.5, 0.75, and 1.0% (w/v) CR). The addition of the CR in SA was based on the methodology described by Tanaka et al.^32^

### 2.3 3D Printing

For the modeling and slicing of the hydrogels, the UltiMaker Cura program was used. For printing, the PronterFace program was installed on the Creality Ender 3 3D printer. The parameters used for printing were as follows: extrusion speed 30 mm/s, travel speed 150 mm/s, wall speed 15 mm/s, flow 58%, layer height 0.6 mm, first layer height 1 mm, two wall fillets, extrusion width 0.4 mm, fill layer thickness 0.6 mm, 0.4 mm nozzle diameter, 10% fill density, extrusion multiplier equal to 1.60 mm/s shrinkage speed, and 2.8 mm top/bottom thickness The models were developed based on the literature.^33^ Figure 1A presents a hydrogel printing model, and Figure 1B shows an example of 3D manufacturing of hydrogel.

**Figure 1:**
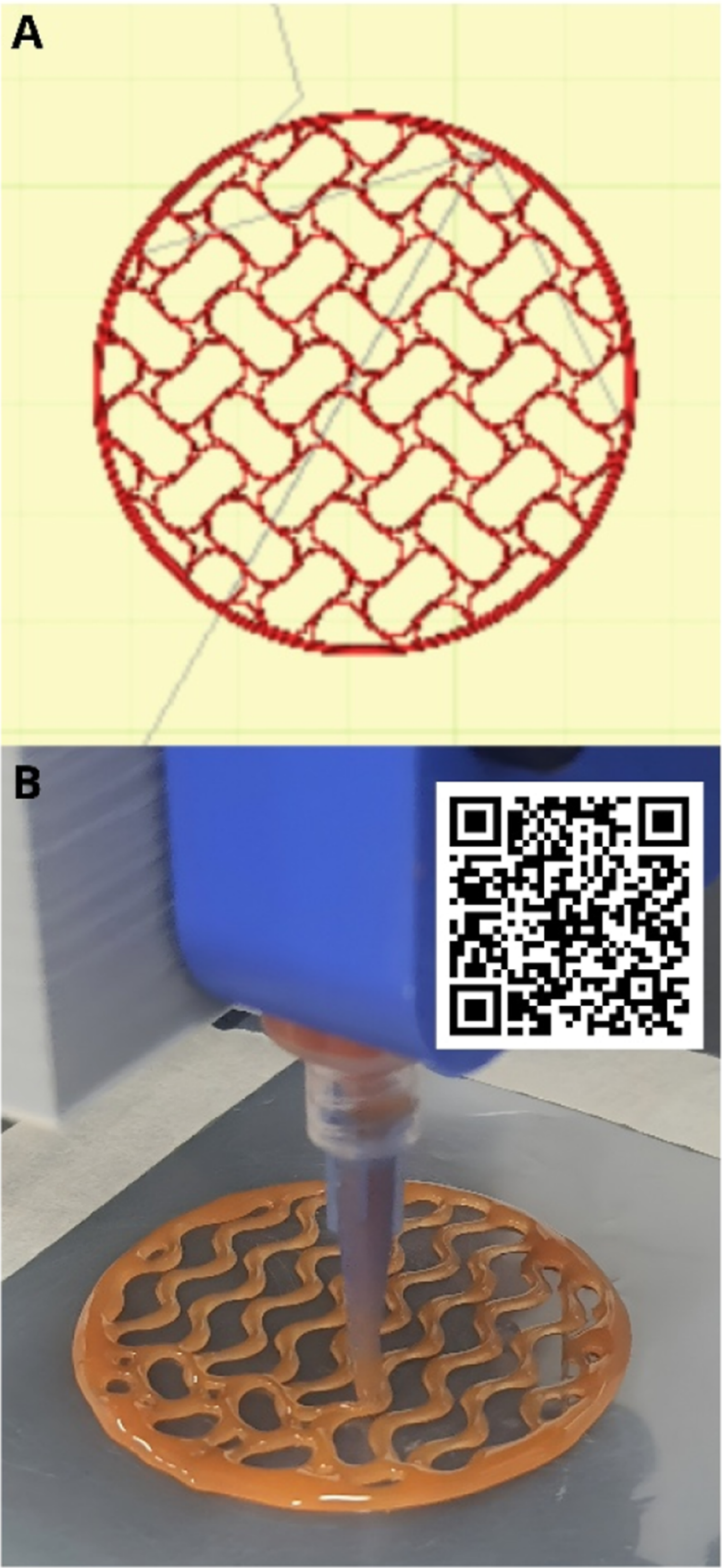
Manufacturing of hydrogels: (A) hydrogel printing model built in the PronterFace program; (B) example of 3D manufacturing of hydrogel. The video can be accessed by QR code or by link:https://youtu.be/iosW4OgoIdQ?si=jhloDW2d8EnM4-Jc.

### 2.4 Cross-Linking of the Printed Hydrogel

For the cross-linking process, the hydrogel was slowly deposited in the Petri dish containing a 1.0% (w/v) calcium chloride solution, remaining for 24 h. It had an area of 49 mm^2^ and an average of 0.3 g of solution. After 24 h of cross-linking, the hydrogel was placed in deionized H_2_O for 24 h for dialysis. For drying, it was placed in the oven at 40 °C for 24 h.

### 2.5 Physico-Chemical Characterizations

#### 2.5.1 Characterization of the Hydrogel Morphology

To study the surface morphology of the hydrogels, a scanning electron microscopy (SEM) technique was used. The preparation samples were those frozen in liquid nitrogen and lyophilized in an Enterprise 2 Terroni freeze-dryer at −55 °C. After this step, the samples were coated with a thin layer of gold to increase electrical conductivity and allocated on a ZEISS electronic microscope (model EVO LS15). The SEM microscope was operated in high-resolution mode to capture detailed images of the surface morphology of the samples, with an acceleration voltage of 7 keV and magnifications of 10,000×. The energy-dispersive X-ray spectroscopy (EDS) technique, coupled to the mentioned ZEISS electronic microscope, was used to identify the chemical elements present in the samples.

#### 2.5.2 Fourier-Transform Infrared Spectroscopy

Fourier-transform infrared spectroscopy (FTIR) was employed to analyze the functional groups and possible interactions between the materials forming the hydrogels. The hydrogel granules were ground, mixed with KBr, and pressed to form pellets. Spectra were obtained using a Nicolet—NEXUS 670 FTIR spectrophotometer, covering the spectral range from 4000 to 400 cm^–1^, accumulating 128 scans with a resolution of 2 cm^–1^.

#### 2.5.3 Thermogravimetric (TG) Analysis

TG experiments were conducted by using a TA Instruments SDT Q600 thermal analyzer. Approximately 8 mg of material was placed in a platinum sample holder and heated from room temperature to 900 °C under a nitrogen atmosphere at a flow rate of 100 mL/min and a heating rate of 10 °C/min.

#### 2.5.4 X-ray diffraction

The hydrogels were ground, placed on glass slides, and positioned in a Shimadzu XRD-6000 X-ray diffractometer equipped with Cu K_α_ radiation (λ = 0.154 nm). The diffraction curves were obtained using the following parameters: voltage of 30 kV; current of 40 mA; 2θ angular range from 5° to 50°, and a scanning speed of 2°/min.

#### 2.5.5 Swelling Degree

The water absorption capacity of the hydrogels with several CR concentrations (0.1, 0.25, 0.5, and 1.0% (w/v)) was evaluated through the swelling degree (SD %) at different time intervals (0.5 to 72 h). Dry samples were immersed in 50 mL of distilled water, and the excess of water superficial moisture was removed with filter paper prior to each measurement. All experiments were performed in triplicate.

The swelling cycle, which was repeated seven times, consisted of three steps: water diffusion, hydrogel expansion, and swelling equilibrium. For each cycle, samples were dried at 40 °C for a minimum of 24 h. Additionally, a separate experiment was carried out to investigate the influence of pH (2, 4, 6, 8, and 10) on water absorption using the same procedure as described above, except that the samples were immersed in aqueous solutions adjusted to the corresponding pH levels. Each study was also repeated in triplicate to ensure the data reliability.

The swelling degree (SD) was calculated using eq 1:

1 $$SD\;\left(\%\right)=\left(\left(M_{swollen}-M_{dry}\right)/M_{dry}\right)\times 100$$

where *M*_swollen_ and *M*_dry_ represent the mass of the hydrogel in swollen and dry states, respectively.

#### 2.5.6 Mechanical Analysis

The alginate and CR-containing hydrogels were evaluated by a tensile test using the TA.XTExpress texturometer, following the standards ASTM D 790-03. The wet samples were subjected to tensile loading with a load cell with a capacity of 5 kg and a constant velocity of 0.5 mm/min until failure (*n* = 5). The height and the diameter were calculated by averaging between 5 different points of each sample. The stress–strain curves were used to determine the elongation, maximum stress at breaking, and modulus of elasticity.^34^

#### 2.5.7 Release Profile of CR

Ultraviolet–visible (UV–vis) spectroscopy was employed to evaluate the release of CR from the tested hydrogel matrices using a Shimadzu UV-2600 spectrophotometer calibrated with a reference solution (50% deionized water and 50% absolute ethanol, v/v). The analyses were conducted between 190 and 750 nm using deuterium and tungsten lamps to cover both the UV and visible ranges. Around 23 mg of hydrogels were immersed into 50 mL of the release medium, and the absorbance readings were taken at various time intervals (1 to 72 h). The quantification of CR was based on a calibration curve constructed at 430 nm, with standards ranging from 0.00015 to 0.0037 mg/L. For absorbances within this range, linear interpolation based on the Lambert–Beer Law was applied. For values above this range, linear extrapolation was used based on the original regression equation. Final concentrations were obtained using both approaches, ensuring data reliability within the method’s limits.

### 2.6 Biological Assays

#### 2.6.1 Antimicrobial Activity

The disk diffusion assay of the hydrogels against *S. aureus* (ATCC 27664 and ATCC 19095) was evaluated using the agar disk diffusion method, following the protocol recommended by the Clinical and Laboratory Standards Institute, 2015, with some adaptations. Bacterial suspensions were prepared at a concentration of 10^8^ CFU/mL. The culture medium used was Müller-Hinton Agar, HiMedia Laboratories. On the previously surface-inoculated medium, 7.0 mm diameter disks of the samples containing 0.5% and 1.0% (w/v) of CR were inserted. The samples were preswollen in sterile distilled water for 24 h. The positive control consisted of culture medium with 30 mg of chloramphenicol disks (Laborcin), and the negative control consisted of medium with treatment disks without CR. As a standard, hydrogels in disk format without CR were used. The Petri dishes were incubated at 37 °C for 24 h. Afterward, the diameters of the inhibition zones formed were measured and expressed in millimeters. The test was performed in a triplicate.

#### 2.6.2 Cell Viability

The cytotoxicity assay was conducted using L929 murine fibroblasts obtained from ATCC, provided by the School of Dentistry of Araraquara/UNESP. The cells were cultured in DMEM supplemented with 10% fetal bovine serum (FBS) and antibiotics (penicillin 100 U/mL; streptomycin 0.1 mg/mL) and incubated at 37 °C with 5% CO_2_. After two passages, the cells (2 × 10^4^ cells/well) were seeded into 96-well plates and incubated for 24 h. Simultaneously, treatments with 0, 0.5%, and 1.0% (w/v) CR were prepared according to ISO 10993-12, using 1.0 cm^2^ of each film sterilized by UV radiation (30 min) and immersed in 3.0 mL of DMEM + 10% FBS for 24 h at 37 °C under agitation. After cell monolayer establishment, the eluates were applied (200 μL/well) and incubated for another 24 h. Then, the cells were washed with PBS and treated with 100 μL of MTT (1 mg/mL), followed by incubation for 3 h at 37 °C in the dark. After formazan crystal formation, MTT was removed and the crystals were dissolved in 50 μL of isopropyl alcohol. Absorbance was measured in a microplate spectrophotometer at 570 nm. The negative control consisted of cells treated only with DMEM + 10% FBS. Experiments were performed in triplicate in three independent assays, and cell viability was calculated based on the average absorbance relative to the survival control (100%), following ISO 10993-5.

### 2.7 Kinetic Correlation Analyses

The release kinetics of *C. longa* L. (CR) from the hydrogel matrices were analyzed based on the UV–vis spectroscopy data, where curcumin concentration was calculated from absorbance values at 430 nm. The experimental release profiles (mg/L vs time) were fitted to the following classical kinetic models.

#### 2.7.1 Zero-Order Model

*Q*_t_ = *Q*_0_ + *k*_0_. *t*, where *Q*_t_ is the amount of CR released at time *t*, *Q*_0_ is the initial amount (usually zero), and *k*_0_ is the zero-order release rate constant.

#### 2.7.2 First-Order Model

ln *Q*_t_ = ln *Q*_0_ + *k*_1_. *t*, where *Q*_t_ is the amount of CR remaining and *k*_1_ is the first-order release constant.

#### 2.7.3 Higuchi Model

*Q*_t_ = *k*_H_. *t* ^1/2^, assuming diffusion-controlled release, where k_H_ is the Higuchi dissolution constant.

#### 2.7.4 Korsmeyer–Peppas Model

ln (*M*_t_/*M*_∞_) = ln *k* + *n* ln *t*, where *M*_t_/*M*_∞_ is the fraction of CR released at time *t*, *k* is a release constant, and *n* is the diffusion exponent:

- *n* ≤ 0.5: Fickian diffusion.
- 0.5 < *n* < 1: anomalous (non-Fickian) transport.
- *n* = 1: zero-order release.

## 3 Results and Discussion

### 3.1 Ink SA/CR Preparation

From the SA/CR solution formed, it was possible to verify the successful incorporation of CR during the cross-linking process of SA, without precipitation or phase separation until printing. Additionally, after preliminary studies, optimal viscosity conditions for printing were achieved. Color change in the solution was also observed during the process, varying according to the CR concentrations added, showing the solubilization of CR in the polymer matrix.

### 3.2 Cross-Linking of the Printed Hydrogel

Printability was successfully achieved, allowing for the precise visualization of the predesigned structure. The hydrogels, regardless of CR incorporation, maintained their structures after printing.

After the cross-linking procedure, it was verified that the printed model’s structure remained unchanged, even after the swelling process. The hydrogels expanded progressively during swelling, as expected. For the hydrogels without CR, an approximate increase of 8.0 mm, in relation to the dried state, was observed after the swelling process. It was also observed that the hydrogels containing 1.0% (w/v) CR showed less expansion, around 4.3 mm, confirming the physical cross-linking action of CR on the polymer matrix.

### 3.3 Physico-Chemical Characterizations

#### 3.3.1 Characterization of the Hydrogel Morphology

Although the hydrogel surfaces, even those of the pure CA control (Figure 2A,B), did not show a direct effect on water absorption, the swelling degree remained unchanged despite variations in the CR concentration.

**Figure 2:**
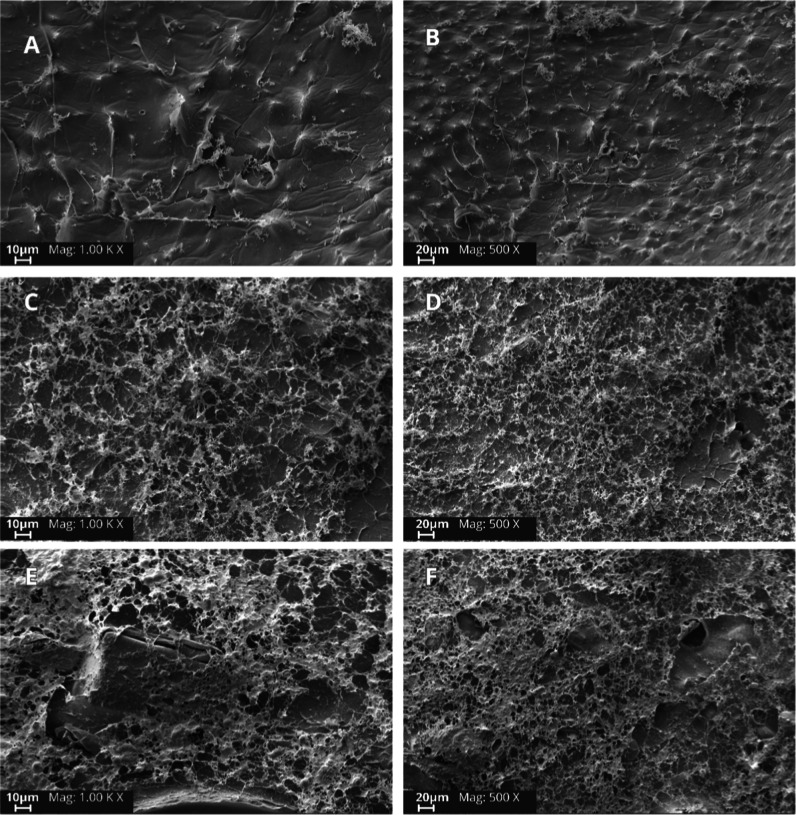
SEM images of (A,B) CA hydrogel; (C,D) hydrogel 0.5% (w/v) CR; (E,F) hydrogel 1.0% (w/v) CR.

The morphological evaluation revealed clear differences among the formulations. The pure alginate hydrogel displayed a relatively smooth and homogeneous surface, consistent with its uniform gel-forming properties (Figure 2A,B). In contrast, the incorporation of higher CR concentrations led to a noticeable increase in surface roughness compared with the control and lower CR-loaded samples (Figure 2C–F). This effect can be attributed to the partial aggregation of CR within the hydrogel matrix. In addition, solid fragments of CR were observed, particularly in the 1.0% (w/v) formulation (Figure 2E), suggesting that a higher CR loading exceeded the solubilization and integration capacity of the alginate network. Similar morphological alterations, including rougher surfaces and visible curcumin domains at higher loadings, have been reported for alginate–curcumin systems.^35,36^

From the results (Table S1 and Figure S1 of Supporting Information), it is possible to observe in the EDX spectrum of pure CR only the elements C and O, as expected. In relation to the EDX of CA hydrogels, in addition to the elements C, O, and Na from the chemical structure of sodium alginate, Cl and Ca elements originating from the physical cross-linking process of sodium alginate were observed. These same elements were observed in the hydrogels containing CR. Although this technique is not a quantitative analysis, the results indicated that the presence of CR retained higher amounts of the elements Na, Cl, and Ca during the cross-linking process. This indicates that the CR may have hindered the process of cross-linking the alginate as well as hindering the dialysis process. Despite this effect, no losses were observed in the other properties of the scaffolds due to the presence of CR.

#### 3.3.2 Fourier Transform Infrared Spectroscopy

The spectrum for 1.0% (w/v) CR (Figure 3A) exhibited the same characteristic bands observed in the hydrogel without CR, indicating that the polymeric network structure was preserved. Only slight changes were detected in specific regions: the band at 1617 cm^–1^, associated with carboxylate stretching, and the band at 1425 cm^–1^ related to C–O and C–C vibrations displayed slight intensity variations compared to pure SA, suggesting overlapping with CR signals. Moreover, the broad band at 3411 cm^–1^, attributed to the O–H stretching, showed a slight shift, indicating the formation of new hydrogen bonds. In addition, two distinct bands at 1283 cm^–1^ (C–O aromatic stretching) and 1510 cm^–1^ (C–C stretching vibrations of the benzene ring in curcumin) were clearly detected, confirming the incorporation of CR into the hydrogel matrix. These findings are further corroborated by the color change observed in the samples after CR incorporation,^23,37,38^ and together, they support the intermolecular interactions proposed in the schematic model by us (Figure 3B).

**Figure 3:**
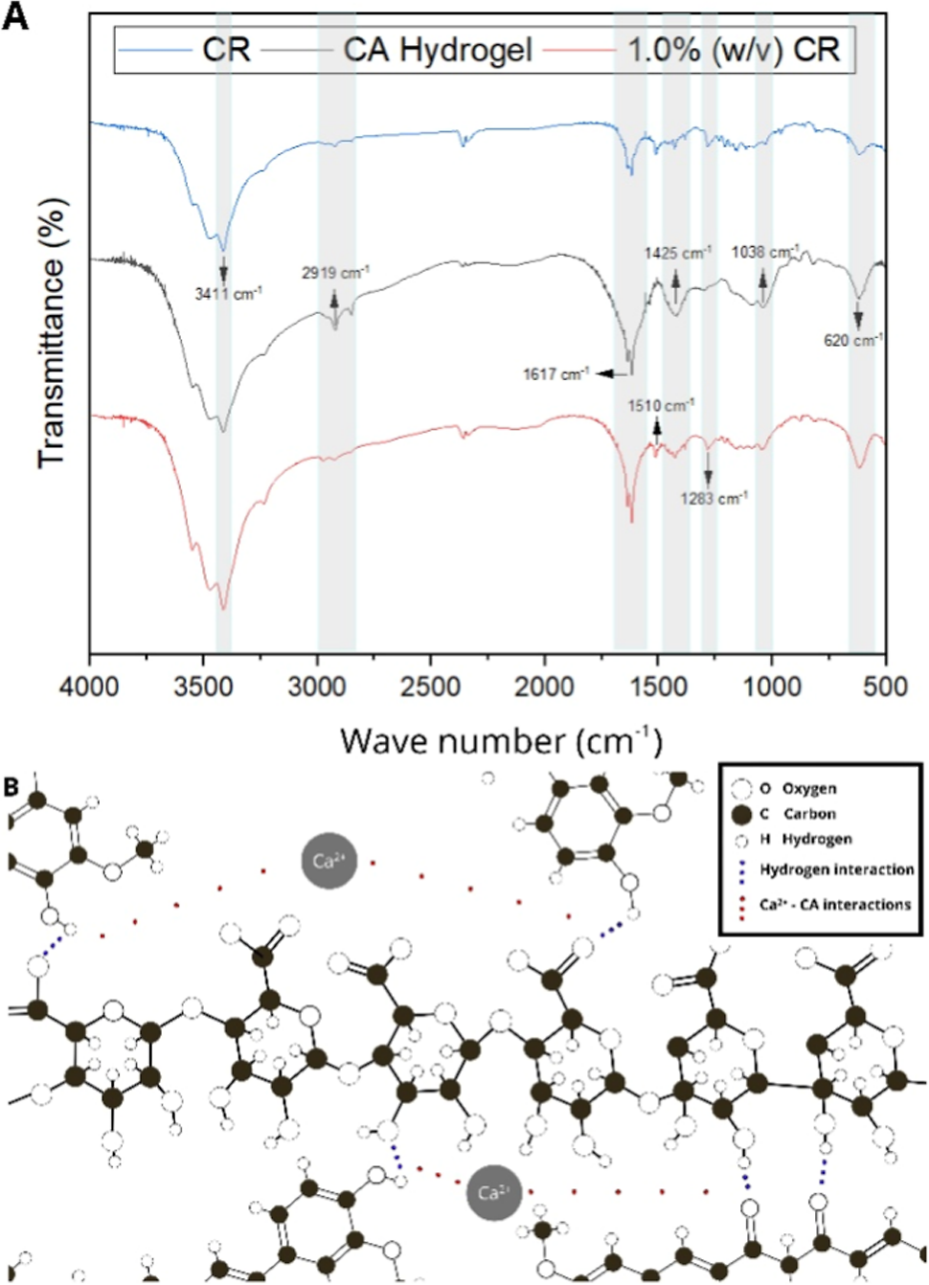
Characterization assessment of the studied hydrogels. (A) Spectra obtained by Fourier-transform infrared absorption spectroscopy (FTIR) of hydrogels containing 0.5% and 1.0% (w/v) of curcumin and pure curcumin (CR); (B) proposed mechanism illustrating possible interaction points between curcumin (CR) and calcium alginate (CA) molecules.

#### 3.3.3 Thermogravimetric (TG) and Differential Thermogravimetric (DTG) Analyses

Figure 4 shows the TG (Figure 4A) and DTG (Figure 4B) curves, where it can be observed that the presence of CR increased the thermal stability of the CA matrix, as the CR-containing samples showed lower mass loss at intermediate and final temperatures as well as an increase in the initial degradation temperature of the second thermal event (T_onset_ or *T*_d_). This result indicates that CR may act as a thermal stabilizer, and it is consistent with previous studies, which highlight the role of CR in enhancing thermal resistance and reducing the intensity of decomposition at each stage.^39,40^

**Figure 4:**
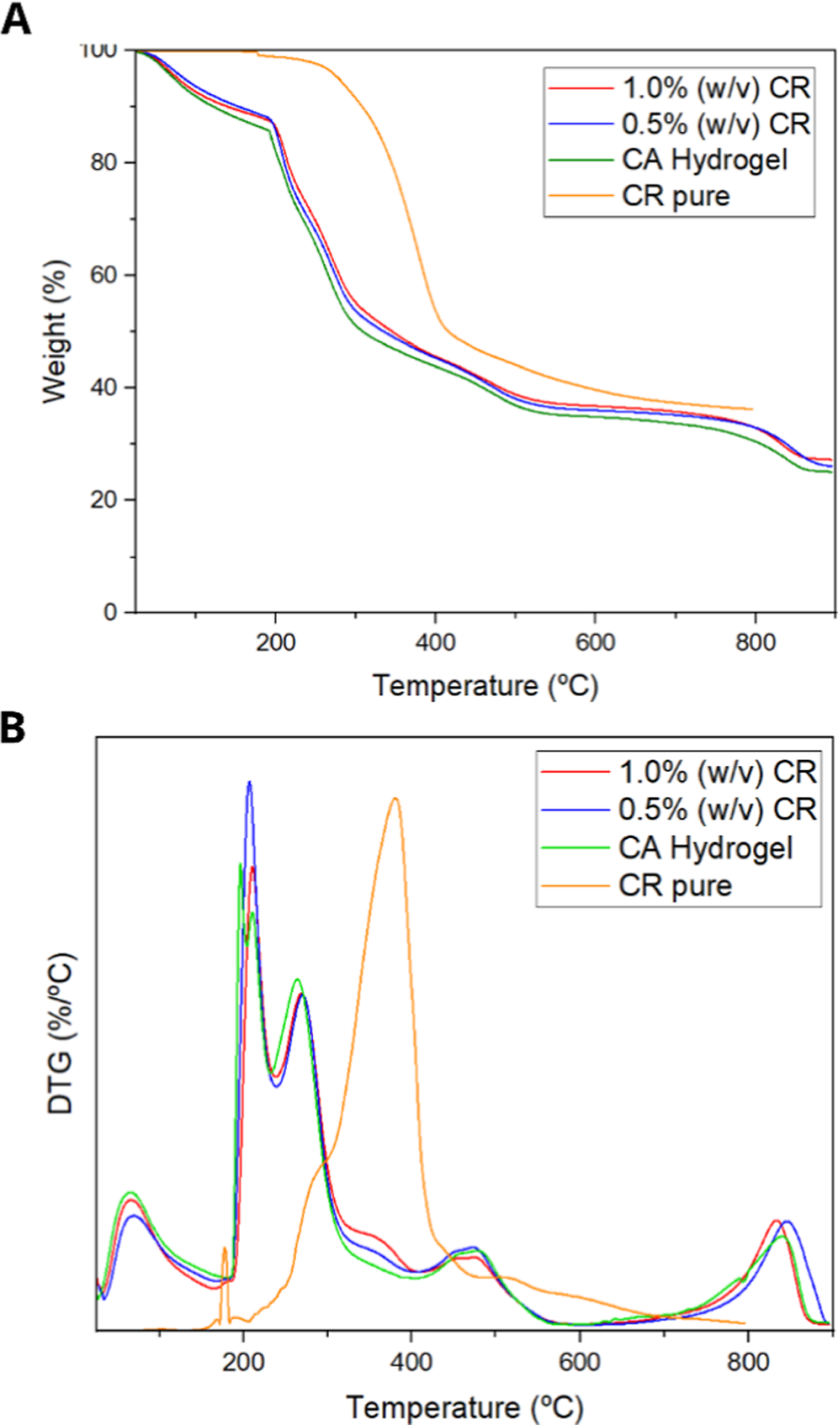
(A) Thermogravimetric spectra of hydrogels containing 0.0%, 0.5%, and 1.0% (w/v) CR and pure CR as a function of temperature (°C); (B) derivative thermogravimetric spectra of hydrogels containing 0.0%, 0.5%, and 1.0% (w/v) CR and pure CR as a function of temperature (°C).

When exposed to high temperatures, materials can undergo chemical or physical modifications, such as volatilization, dehydration, decomposition, oxidation, and fusion. In pure CR, a first thermal event was observed between 172.7 and 184.3 °C (maximum temperature—*T*_m_-at 178.3 °C), which was attributed to the dehydroxylation of –OH groups with the elimination of water molecules.^39^ The second event, between 184.3 and 473.3 °C (with maximum at 380.8 °C), corresponded to the decomposition of the aromatic rings of curcumin, acetaldehyde ketone, and phenoxyacetic acid,^40^ leaving a residue of 36.2% due to nonvolatile intermediates, with the formation of amorphous carbon.^39^

For CA-based samples, five main thermal events were identified. The first, between ∼27.9 and 167.7 °C, was associated with the loss of adsorbed water and volatile compounds. da Silva Fernandes et al.^41^ reported the dehydration of CA between 38 and 174 °C, while Zhao et al.^42^ observed water elimination around 120 °C, with mass loss of ∼3.9%. The experimental values confirmed this trend, with losses of 10.7 and 13.4% attributed to residual moisture and AC–CR interactions. The *T*_d_ started around 167.0 °C and was related to polymeric degradation of CA, consistent with glycosidic bond cleavage.^42,43^ In samples with CR, this temperature slightly shifted to higher values (188.2 to 208.1 °C), suggesting a stabilizing effect of CR on the CA structure.

The third event, between 238.0 and 402.0 °C, involved intense polymeric degradation with the disruption of CA–Ca^2+^ interactions and the start of CR decomposition,^39^ with overlapping stages between 215 and 354 °C and around 417 °C, while Chen et al.^40^ identified a *T*_m_ at 292 °C. In our study, the *T*_m_ shifted to 275.8 °C in CR-containing samples, indicating an influence of CR on stability. The fourth event, between 381.6 and 600.3 °C, corresponded to CA and CR degradation. This stage involved the breakdown of benzene rings, with a maximum of 468 °C, consistent with our observed *T*_m_ at 481.4 °C.^42^ The fifth event, from 600 to 900 °C, linked to alginate pyrolysis residues and carbonate conversion,^41^ reported CaCO_3_ formation between 600 and 735 °C, while Zhao et al.^42^ indicated stabilization at ∼750 °C. In this work, the maximum reached 845.3 °C, confirming the final thermal degradation and carbonaceous residue formation.

In conclusion, the incorporation of CR enhanced the thermal stability of the CA matrix, as evidenced by reduced mass loss at intermediate and final stages and *T*_d_ of the second thermal event. This finding is consistent with previous studies^39,40^ and highlights the role of CR as a thermal stabilizer. Such behavior is relevant for the potential application of this material as a wound dressing, ensuring greater durability and resistance to conditions such as sterilization (e.g., autoclaving) and storage.

#### 3.3.4 X-ray Diffraction

Pure CR (Figure 5A) exhibited sharp peaks in the range of 10°–30°, indicating a predominantly crystalline structure.^44^ X-ray diffraction (XRD) patterns of the CA revealed a predominance of amorphous regions regarding the structural properties of the matrices (Figure 5B). Diffraction peaks around 17.3° and 24.9° were observed in the CA/CR samples, increasing progressively with the concentration of CR. This is indicative of the increase in the crystallinity of the polymer structure, probably provoked by the new molecular organization induced by the presence of curcumin. A similar effect was reported by da Silva Fernandes et al.^41^ when adding Cloisite-Na^+^ nanoclay in alginate-based nanocomposites. This reorganization may be associated with the formation of interactions between alginate and functional groups from compounds present in CR, which affect the matrix structure and potentially its mechanical and thermal properties, as suggested by Zhang et al.^45^

**Figure 5:**
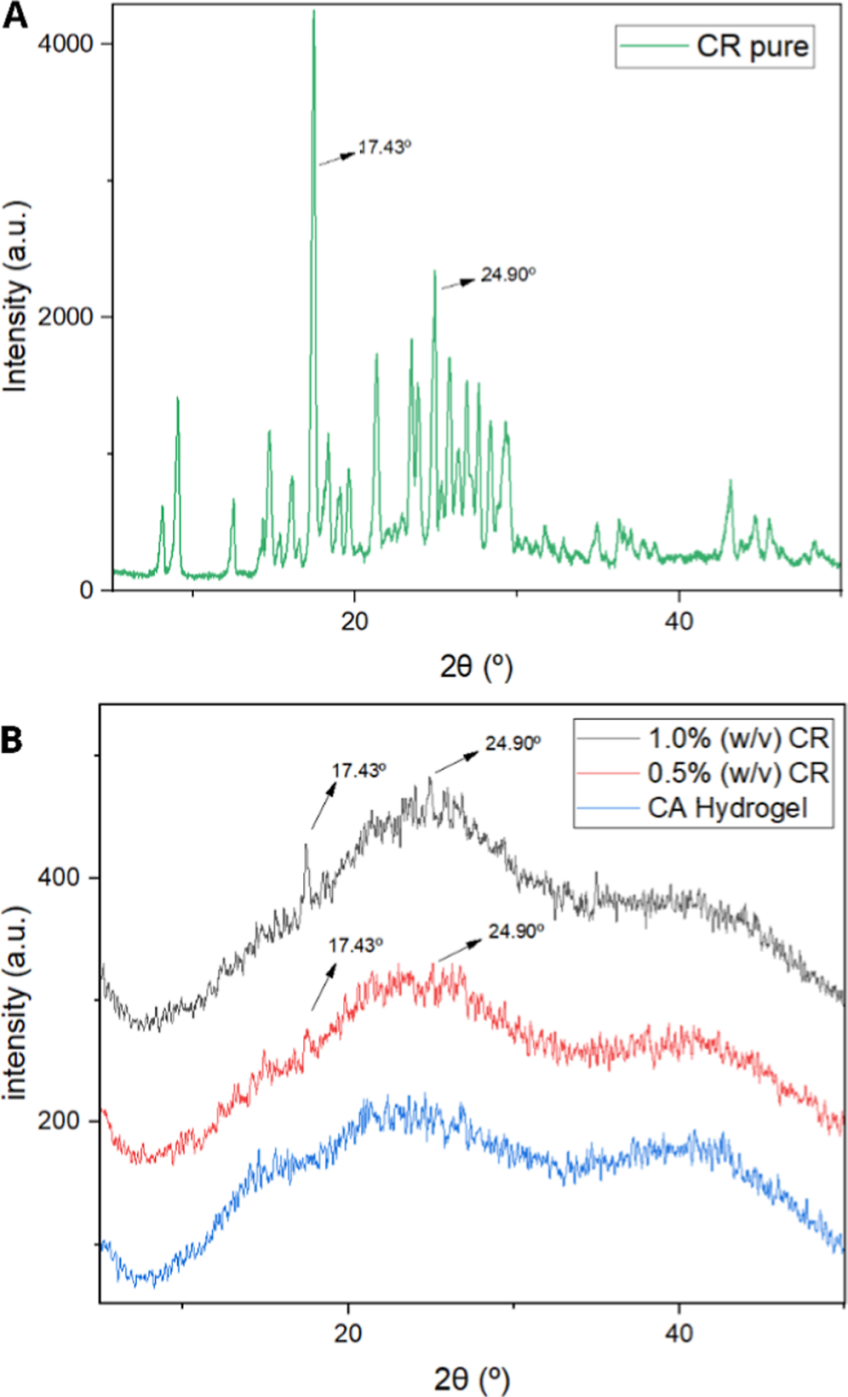
Evaluation of X-ray diffraction characterization. (A) Pure *Curcuma longa* L. (CR); (B) CA Hydrogel, and 0.5% and 1.0% (w/v) CR.

#### 3.3.5 Swelling Degree

The water uptake of the hydrogels for different concentrations of CR was monitored until the equilibrium stage (Figure 6A). All matrices rapidly absorb water and reached equilibrium in approximately 8 h, demonstrating suitability for applications that require quick water absorption and stable behavior over time. After 48 h (Figure 6B), the swelling degree remained relatively stable across all CR concentrations, averaging around 160%. This suggests that CR, even at concentrations up to 1.0% (w/v) CR, does not significantly impact the long-term water absorption capacity. In this way, the hydrogels maintained their performance independently of the amount of CR incorporated, indicating good stability and reproducibility, which are essential for biomedical or cosmetic applications where consistency is critical.

**Figure 6:**
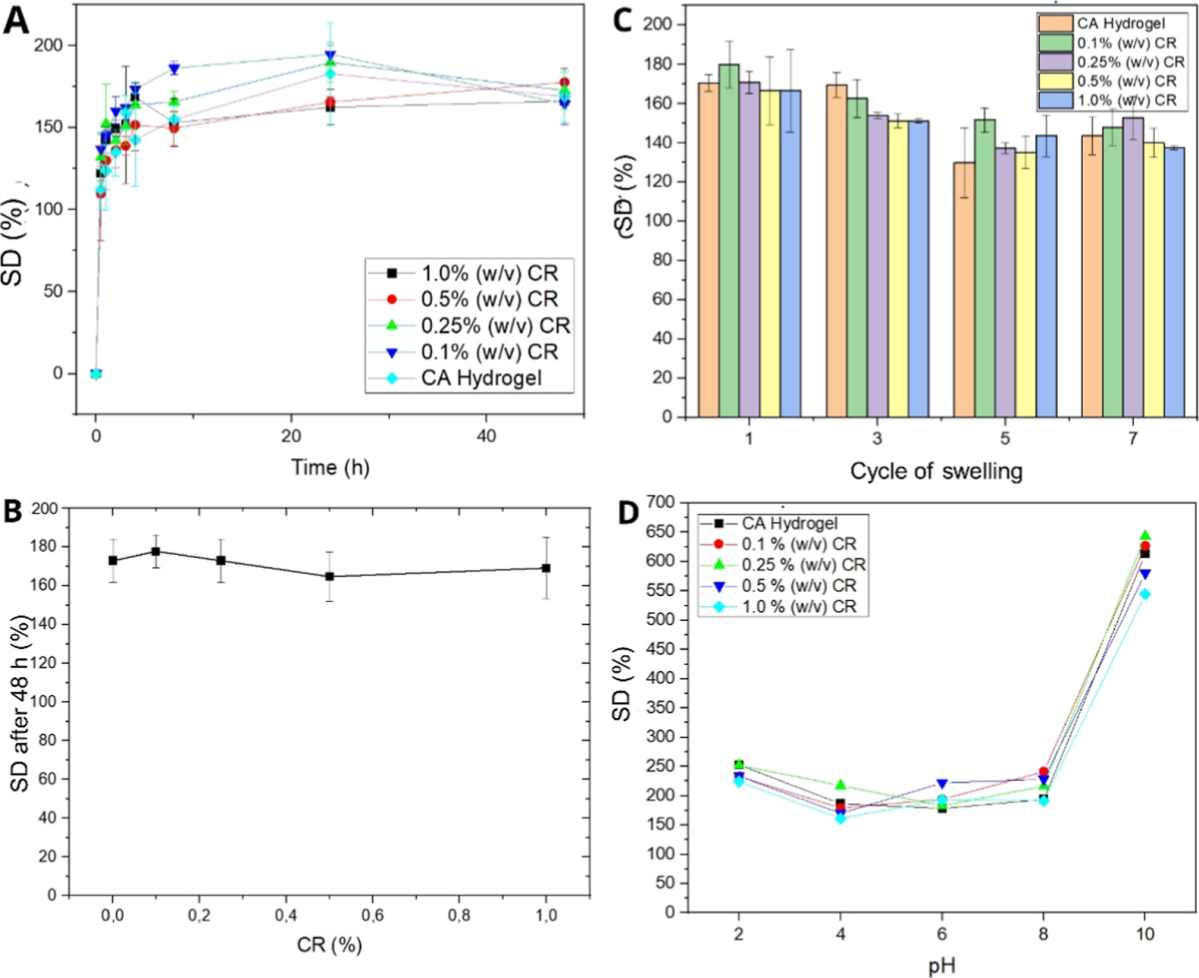
(A) Swelling study of CA hydrogel and hydrogels containing 0.1%, 0.25%, 0.5%, and 1.0% (w/v) CR; (B) swelling study of hydrogels containing CR over 48 h by CR concentration (CR); (C) swelling cycles performed with CA hydrogels 0.1%, 0.25%, 0.5%, and 1.0% (w/v) CR over 48 h; (D) swelling study of CA hydrogels 0.1%, 0.25%, 0.5%, and 1.0% (w/v) CR under different pH conditions.

The cyclic swelling degree behavior (Figure 6C) demonstrated promising results, where the hydrogels retained most of their swelling capacity over seven consecutive cycles. These findings suggest that the CA/CR hydrogels can be reused multiple times without a substantial loss of performance, reinforcing their applicability in systems requiring durability and multiple hydration cycles.

The swelling response under different pH conditions (Figure 6D) revealed interesting behavior. In basic pH conditions (pH 8–10), a significant increase in SD % was observed. This phenomenon can be explained by the chemical nature of alginate, which forms hydrogels through ionic interactions between its carboxylate groups (−COO^–^) and divalent cations such as Ca^2+^. At basic pH, the carboxylic groups are in their deprotonated form (p*K*_a_ ∼ 3.5–4.0), increasing the negative charge density in the polymer network. This leads to stronger electrostatic repulsion between chains, facilitating water intake due to the increase in osmotic pressure.^46,47^

In particular, the combination of low protonation and reduced ionic strength allows the internal osmotic pressure to exceed the elastic forces of the network, leading to progressive swelling as the pH increases.^48^ Moreover, curcumin, the majority compound present in CR, may also undergo structural changes under alkaline conditions, enhancing its interaction with the polymer matrix and modifying the swelling behavior.^49^

#### 3.3.6 Mechanical Analysis

The analysis of the mechanical properties (Table 1) revealed that the bioprinted hydrogels have a maximum stress at breaking of approximately 0.012 MPa and a good elongation capacity, reaching up to 6.05. Notably, no significant differences were observed between the alginate hydrogels and their scaffolds prepared with different concentrations of CR. This satisfactory flexibility is accompanied by a low Young’s modulus (0.0023 to 0.0032 MPa), which indicates that the material has the necessary softness to mimic the viscoelastic behavior of human skin, ensuring that the dressing adapts to dynamic surfaces without causing mechanical trauma to the patient.^50^

**Table 1:** Mechanical Tensile Test of Printed Alginate Hydrogels Containing CR^a^

	maximum stress at breaking (MPa)	strain	Young’s modulus (MPa)
CA hydrogels	0.013 ± 0.003	4.47 ± 0.67	0.0029 ± 0.0006
0.5% (m/v) CR	0.012 ± 0.006	6.05 ± 2.33	0.0032 ± 0.0009
1.0% (m/v) CR	0.012 ± 0.005	5.23 ± 0.56	0.0023 ± 0.0008

a Results expressed as mean (*n* = 5) ± standard deviation.

Although the *layer-by-layer* manufacturing process can introduce interfacial shear zones that influence absolute mechanical strength,^51^ this feature is technically balanced by the porous architecture of the device. The presence of pores and surface irregularities, while reducing structural stiffness compared to dense materials, is a critical functional requirement to optimize exudate control and permeability in chronic wounds.^50^ Therefore, the maintenance of physical integrity after the incorporation of CR, combined with the high deformability observed, validates the potential of these hydrogels as active and resilient supports for the treatment of skin lesions.

#### 3.3.7 Release Profile of CR

The UV–vis spectroscopy technique was used to monitor the release of CR from hydrogel matrices. The absorbance values of the releasing solutions were converted into concentrations using the calibration curve (Figure S2 of Supporting Information). The sample containing 0.5% (w/v) CR had a release profile similar to that observed in the 1.0% (w/v) CR sample but with lower concentrations (Figure 7A,B). A rapid release was observed during the first hours, reaching 0.0041 mg/L after 8 h, becoming slower until the end of the release process (Figure 7C), likely due to medium saturation or reduced concentration gradients that govern diffusion. Accordingly, the 1.0% (w/v) CR sample exhibited faster release during early stages due to the higher initial content of the active compound. The absence of a saturation plateau suggests that further release could occur if the time interval was extended. In this way, when comparing both samples, the 1.0% (w/v) CR hydrogel is more suited for applications requiring fast and higher-dose delivery, especially within the first 8 h.

**Figure 7:**
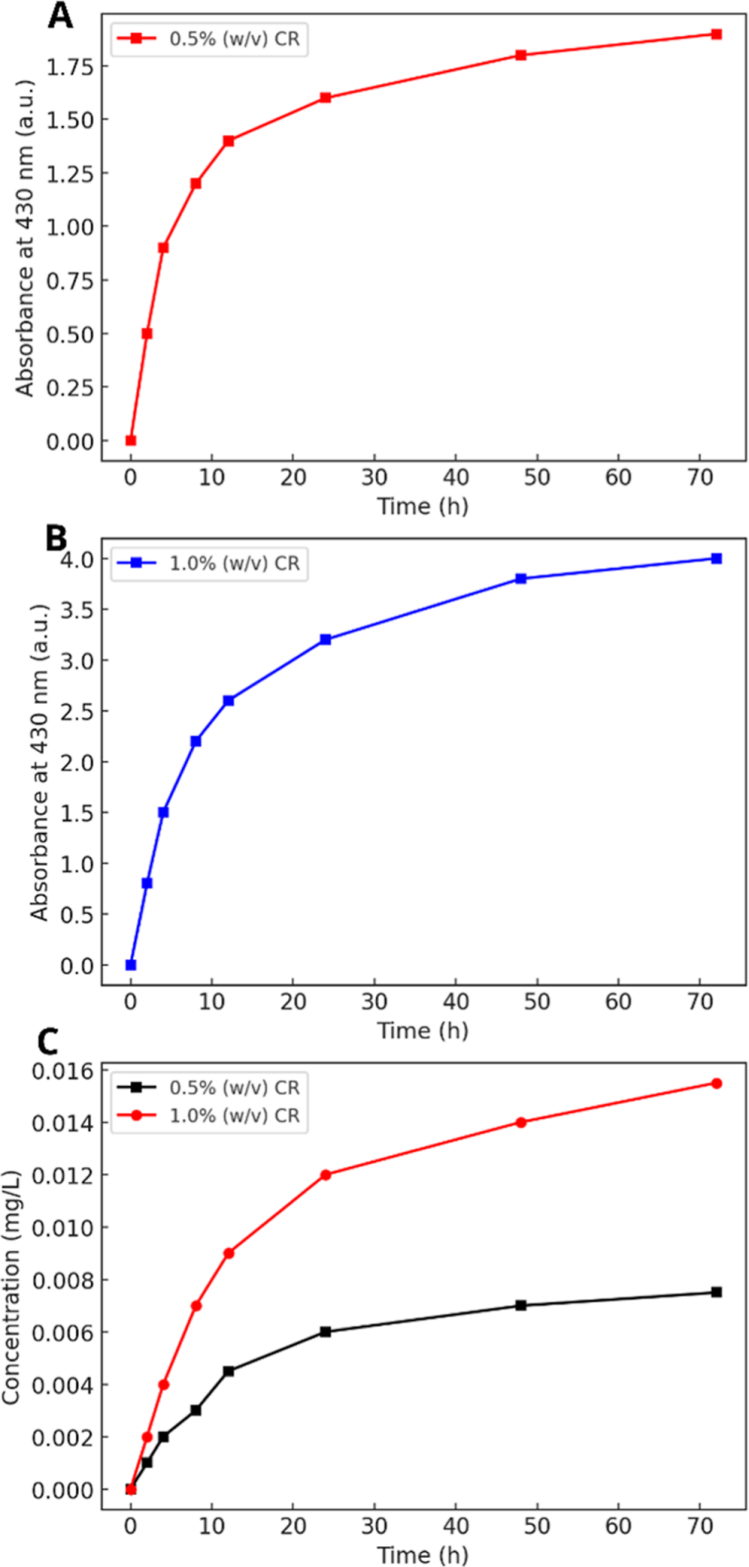
Analysis of *Curcuma longa* L. release from CA hydrogel matrices. Absorbance at 430 nm over time for the (A) 0.5% (w/v) CR sample and the (B) 1.0% (w/v) CR sample. (C) Release profiles expressed as concentration (mg/L) for the 0.5% and 1.0% (w/v) CR samples.

### 3.4 Biological Assays

#### 3.4.1 Antimicrobial Activity

Table 2 presents the results of the disk diffusion assay of the CR-containing samples against the growth of *S. aureus*. Only dressings with 1.0% (w/v) CR demonstrated significant antibacterial activity against Gram-positive strains,^52^ with a reported reduction in the bacterial activity of *E. coli* and *S. aureus* when using films with bacterial cellulose and CR. The bacterial inhibition observed with 1.0% (w/v) CR reinforces its therapeutic potential in bioactive dressings. This effect may be related to curcumin’s ability to interfere with bacterial cell division by inhibiting the FtsZ protein, which is essential for cytokinesis. In addition, CR may prevent biofilm formation by blocking cell communication via quorum sensing.^53,54^

**Table 2:** Antibacterial Activity of CR-Loaded CA Hydrogels on *Staphylococcus aureus* (ATCC 27664 and ATCC 19095)^a^ ^,^ ^b^

treatment	inhibition halo (mm)	
	S. aureus ATCC 27664	S. aureus ATCC 19095
CA hydrogels	n.d	n.d
0.5% (m/v) CR	n.d	n.d
1.0% (m/v) CR	10.77 ± 0.68	12.67 ± 1.53

a Legend: n.d.—not detected.

b Results expressed as mean (*n* = 3) ± standard deviation.

The antibacterial activity ranged from 50 to 70% reduction in cell density, increasing with CR concentration. In a recent study, Abd El-Hady and Saeed (2020) evaluated chitosan-based hydrogels incorporating silver–curcumin nanocomposites, which produced inhibition zones of approximately 13 mm against *S. aureus*, along with a significant reduction in bacterial colony formation.^55^ This data highlights the biomedical potential of such materials in wound healing therapies and tissue engineering.

#### 3.4.2 Cell Viability

The analysis of cell viability using the colorimetric MTT reagent revealed different responses of L929 cells to two different concentrations of CR in the CA hydrogels. As shown in Table 3, the sample 1.0% (m/v) CR exhibited significant cytotoxicity, with cell viability below 70%, specifically 35%. In contrast, the samples of CA hydrogel and 0.5% (m/v) CR maintained cell viability above 70%, reaching 90% and 80%, respectively. These results indicate that while higher concentration of CR was cytotoxic to L929 cells, lower concentration (0.5% w/v CR) was cytocompatible. The CA hydrogel without CR also demonstrated noncytotoxicity toward the tested L929 cells, serving as a positive control to confirm the inherent cytocompatibility of the matrix.

**Table 3:** Cell Viability (%) of L929 Fibroblasts Treated with CR-Loaded Calcium Alginate Hydrogels Using an Indirect MTT Method

CR concentration (% w/v)	cell viability (%)	interpretation
CA hydrogels	90%	noncytotoxic, positive control
0.5% w/v CR	80%	non-cytotoxic
1.0% w/v CR	35%	cytotoxic

The cytotoxic effects observed in CA–CR1.0 may be related to the increased concentration combined with the inherent instability of CR, including high susceptibility to chemical degradation in alkaline aqueous solutions (pH ≥ 7.0) and photodegradation, which can generate oxidative stress and compromise normal cellular function.^56^

Lee et al.^14^ developed a curcumin-loaded nanoemulsion (CUR-NE) gel containing 0.5% CUR, with Capryol 90 as the oil phase, a surfactant mixture of Labrasol:Cremophor RH40 (1:1), and propylene glycol as the cosurfactant. In the in vivo assay, the CUR-NE gel accelerated wound healing, promoting faster re-epithelialization and dermal regeneration, and outperformed both CUR-only and commercial nanosized CUR gels. Curcumin-loaded CMC hydrogels incorporating aloe vera oil were developed.^57^ The prepared formulations were stable and demonstrated no skin irritation effect. Furthermore, ex vivo studies revealed that the hydrogel matrix combined with essential oils effectively enhances topical delivery and local bioavailability of curcumin.

In this context, previous studies highlight the potential of curcumin-loaded dressings to enhance biological responses and accelerate wound healing, owing to their noncytotoxicity and bioactive properties when evaluated under appropriate and controlled conditions.

### 3.5 Correlation Analysis

To better understand the release mechanisms, the data were fitted to four classic kinetic models: zero-order, first-order, Higuchi, and Korsmeyer–Peppas (Table 4). The Korsmeyer–Peppas model showed the best correlation for both 0.5% and 1.0% CR formulations (*R* ^2^ > 0.996), with diffusion exponents of *n* ≈ 0.50 and 0.54, respectively, indicating Fickian to anomalous diffusion-controlled release. The Higuchi model also provided a good correlation (*R* ^2^ ≈ 0.87 versus 0.89), reinforcing the diffusional mechanism. In contrast, the zero- and first-order models displayed lower correlation values (*R* ^2^ ≈ 0.82–0.89), suggesting that they do not fully describe the system. The zero-order, first-order, Higuchi, and Korsmeyer–Peppas models were calculated using the curves of Figure S3 of Supporting Information.

**Table 4:** Kinetic Parameters of *Curcuma longa* L. Release from 0.5% and 1.0% (w/v) Hydrogels

kinetic model	parameters (0.5% CR)	*R* ^2^ (0.5% CR)	parameters (1.0% CR)	*R* ^2^ (1.0% CR)
zero-order	*k* = 4.60^–4^ mg/L·h^–1^; *Q*_0_ = 8.2 × 10^–4^ mg/L	0.835	*k* = 8.42 × 10^–4^ mg/L·h^–1^; *Q*_0_ = 1.2 × 10^–3^ mg/L	0.893
first-order	*k* = 0.134 h^–1^; *Q*_0_ = 1.54 × 10^–3^ mg/L	0.819	*k* = 0.148 h^–1^; *Q*_0_ = 2.45 × 10^–3^ mg/L	0.884
Higuchi	*k*_H_ = 1.46 × 10^–3^ mg/L·h^–1/2^	0.868	*k*_H_ = 2.60 × 10^–3^ mg/L·h^–1/2^	0.893
Korsmeyer–Peppas	*k* = 0.197; *n* = 0.50	0.997	*k* = 0.155; *n* = 0.54	0.996

To further understand the relationships between formulation parameters and biological responses, a Pearson correlation analysis was performed among the CR concentration, curcumin release, cell viability, and antimicrobial activity. As illustrated in Figure 8, a strong negative correlation was observed between CR concentration and cell viability (*r* = −0.94) as well as between CR release and viability (*r* = −0.94). Likewise, there was a strong positive correlation between CR concentration and antimicrobial activity (*r* = 0.87) and between CR release and antimicrobial activity (*r* = 0.87). Notably, antimicrobial activity showed an almost perfect inverse correlation with cell viability (*r* = −0.99), reinforcing the dose-dependent trade-off between antibacterial efficacy and cytotoxicity.

**Figure 8:**
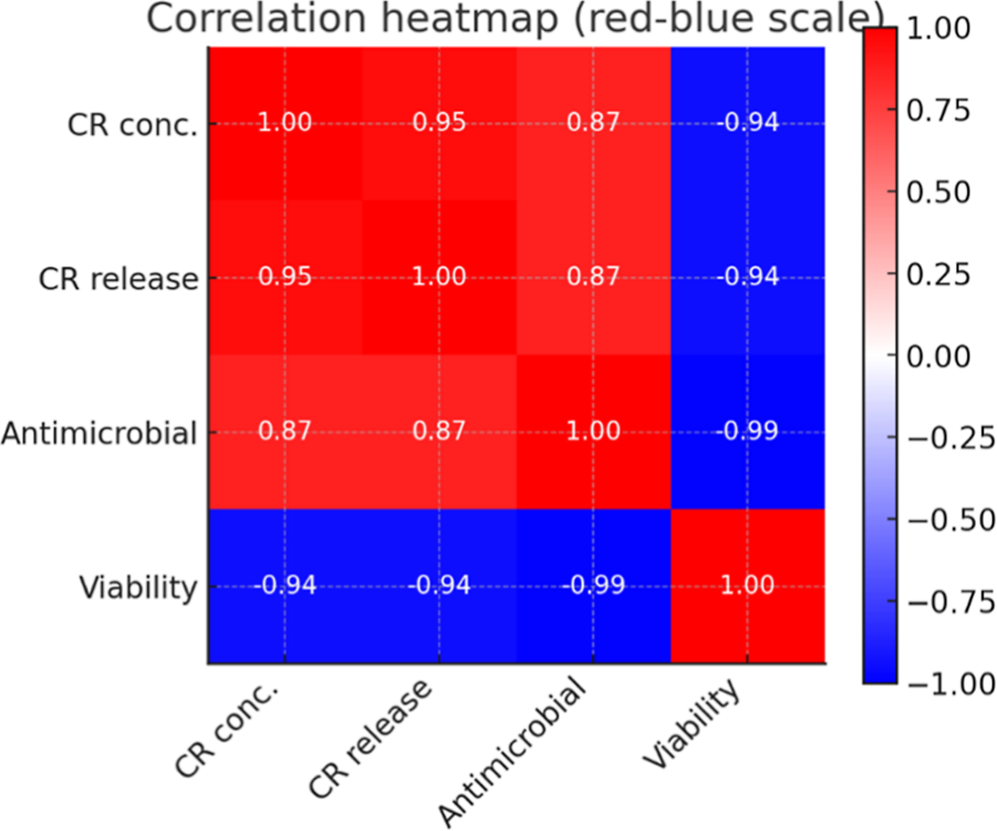
Heatmap of Pearson correlation coefficients among CR concentration, CR release, antimicrobial activity, and cell viability. Strong positive correlations were observed between CR concentration/release and antimicrobial activity (*r* = 0.87 – 0.95), whereas both variables showed negative correlations with cell viability (*r* = −0.94). Antimicrobial activity exhibited the strongest negative correlation with viability (*r* = −0.99), confirming the dose-dependent effect of CR.

These results indicate that higher CR concentrations and greater release enhance antibacterial performance but significantly reduce cell viability. Conversely, the 0.5% (w/v) CR hydrogel maintained adequate biocompatibility (80% viability) but lacked a detectable antimicrobial effect. The 1.0% (w/v) CR sample, in contrast, provided strong antibacterial activity but with high cytotoxicity.

Together, these findings highlight the importance of balancing the CR content in hydrogel matrices: while higher doses are more effective against bacteria, they compromise cell biocompatibility. Tailoring the CR concentration allows the system to be adjusted according to the desired biomedical application, ranging from wound dressings prioritizing antimicrobial action to scaffolds requiring a cell-friendly environment.

## 4 Conclusion

This study successfully developed and characterized 3D-bioprinted hydrogels composed of calcium alginate and *Curcuma longa* L. (CR), demonstrating their promising potential for use as bioactive wound dressings. Structural and thermal analyses confirmed the stability and integration of CR into the hydrogel matrix, while swelling experiments showed that water absorption remained consistent (∼160%) regardless of the CR concentration, indicating excellent reproducibility and hydration performance. No variation in the mechanical properties of the alginate-based scaffolds was observed by the addition of CR. The observed cytotoxic effects at higher CR concentrations might also be attributed to the synergistic action of curcuminoids and other phytochemicals, such as flavonoids, present in the turmeric extract, which can interfere with cellular homeostasis.

The release profile of CR was concentration-dependent, with the 1.0% (w/v) hydrogel exhibiting a faster and higher release compared with the 0.5% (w/v) formulation. Kinetic modeling demonstrated that the Korsmeyer–Peppas model provided the best fit (*R* ^2^ > 0.99), with release exponents *n* ≈ 0.50–0.54, indicating Fickian to anomalous diffusion as the main release mechanism. The Higuchi model also showed good correlation (*R* ^2^ ≈ 0.86–0.89), further supporting a diffusion-controlled process, whereas the zero- and first-order models showed lower correlation values, suggesting that they do not adequately describe the system.

Biological evaluations showed that the 1.0% (w/v) CR hydrogel exhibited significant antibacterial activity against *Staphylococcus aureus* but was cytotoxic to L929 fibroblasts (35% of viability). In contrast, the 0.5% (w/v) CR hydrogel maintained cytocompatibility (80% viability) while still enabling the sustained release of the active compound. Correlation analysis revealed a positive correlation between CR concentration/release and antibacterial activity (*r* = 0.87) and a negative correlation with cell viability (*r* = −0.94 to −0.99), indicating the dose-dependent behavior of the system.

Overall, these findings highlight the system’s versatility and the importance of carefully optimizing the CR concentration. The 0.5% (w/v) CR formulation appears to be particularly promising, combining controlled release, thermal stability, reusability, and biocompatibility. CR presents intrinsic limitations in hydrogel-based systems, including low aqueous solubility, pH-dependent instability, and pronounced photodegradation, which can compromise the bioavailability and sustained release in epithelial tissues. Strategies to overcome these limitations include the incorporation of nanocarriers (e.g., liposomes, polymeric nanoparticles, cyclodextrin complexes) and the use of antioxidants or UV-protective agents for curcumin to improve its solubility and photostability.

## Supplementary Material



## Notes

EDX analysis and mapping used for identification of the chemical elements, calibration curve used for the quantification of the controlled release of the active compound, and curves and parameters of kinetic release analyses (applied mathematical models) (PDF)
