# Supplementary material for: 3D-Bioprinted Hydrogels Based on Calcium Alginate and Turmeric (Curcuma longa L.): Comprehensive Structural, Functional, and Biological Evaluation for Advanced Wound Healing Applications
Source: ACS Omega. 2026 Mar 16;11(12):19740–51. doi: 10.1021/acsomega.6c00044 (PMC13044693; doi:10.1021/acsomega.6c00044)
Supplement: Supplementary file 1 [file ao6c00044_si_001.pdf]

# 3D Bioprinted Hydrogels Based on Calcium Alginate and Turmeric (*Curcuma longa* L.): Comprehensive Structural, Functional, and Biological Evaluation for Advanced Wound Healing Applications

Rafaela Prediger dos Anjos[1,*], Paula de Abreu Fernandes[2], Hernane da Silva Barud[2], Marina de Lima Fontes[2], Marília Gonçalves Cattelan[3], Marcia Regina de Moura[1], Fauze Ahmad Aouada[1,#]

[1]*Group of Composites and Hybrid Nanocomposites (GCNH), São Paulo State University (UNESP), School of Engineering, Ilha Solteira, SP, Brazil.* [2] *Laboratory of Biopolymers and Biomaterials (BioPolMat) - Uniara.* [3] *Food Microbiology Laboratory, São Paulo State University (UNESP).*

**Corresponding author Rafaela Prediger dos Anjos, Me., Phone number: + 55 18 3743-1195*

*e-mail address: rafaela.prediger@unesp.br*

*#Corresponding author Fauze Ahmad Aouada, Ph.D., Phone number: + 55 18 3743 -1195*

*e-mail address: fauze.aouada@unesp.br*

**Table S1 -** EDX analysis for CR, CA hydrogel, and hydrogels with 0.5 and 1.0 % (w/v) CR.

| | (% wt) | | | |
|---|---|---|---|---|
| Chemical Element | CR pure | CA Hydrogel | Hydrogel 0.5% (w/v) CR | Hydrogel 1.0% (w/v) CR |
| C | 68.66 | 43.38 | 36.74 | 34.24 |
| O | 31.34 | 48.87 | 44.33 | 37.95 |
| Na | * | 0.09 | 1.12 | 1.09 |
| Cl | * | 0.14 | 7.77 | 12.57 |
| Ca | * | 7.52 | 10.04 | 14.15 |

* undetected element.

**Figure S1 -** EDX mapping (A) CR pure; (B) CA Hydrogel; (C) Hydrogel 0.5% (w/v) CR; (D) Hydrogel 1.0% (w/v) CR.

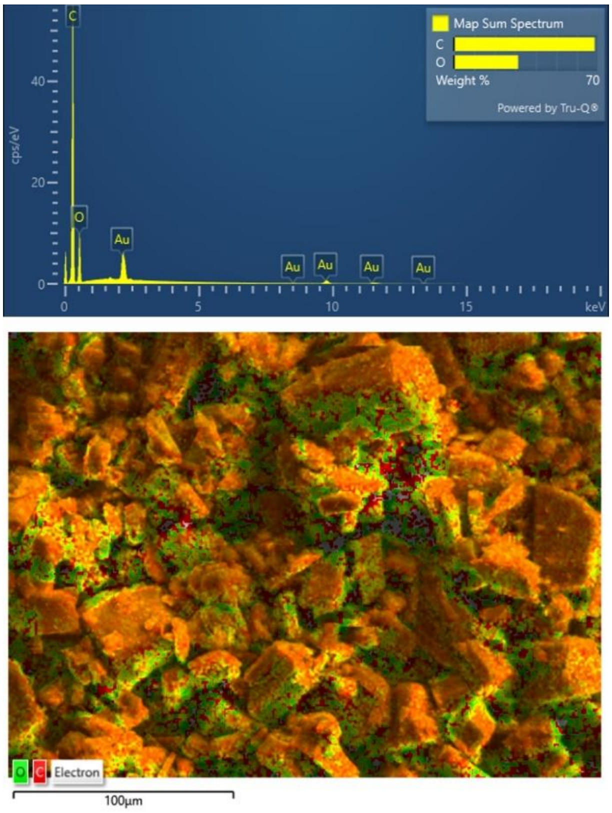


(a)

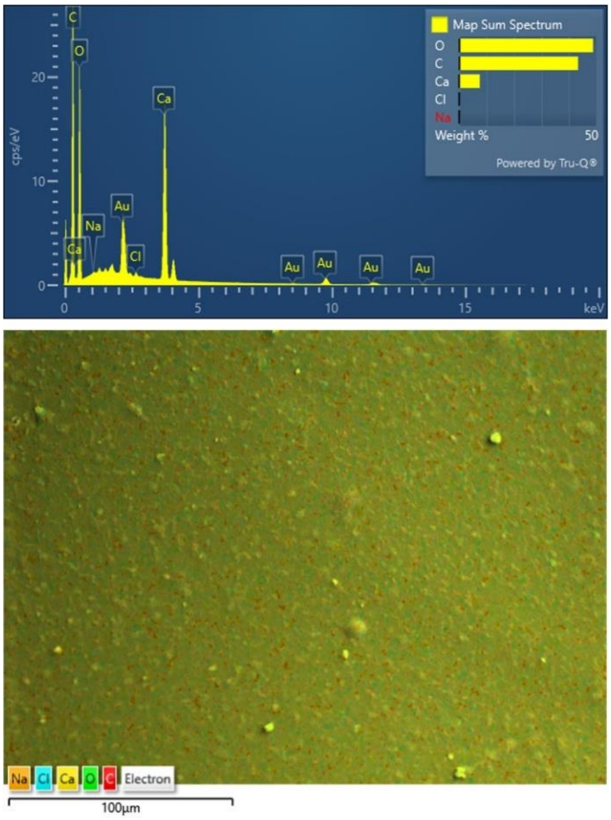
Map Sum Spectrum
O
C
Ca
Cl
Na
Weight %
50
Powered by Tru-Q®
cps/eV
keV
Na Cl Ca O C Electron
100µm

(b)

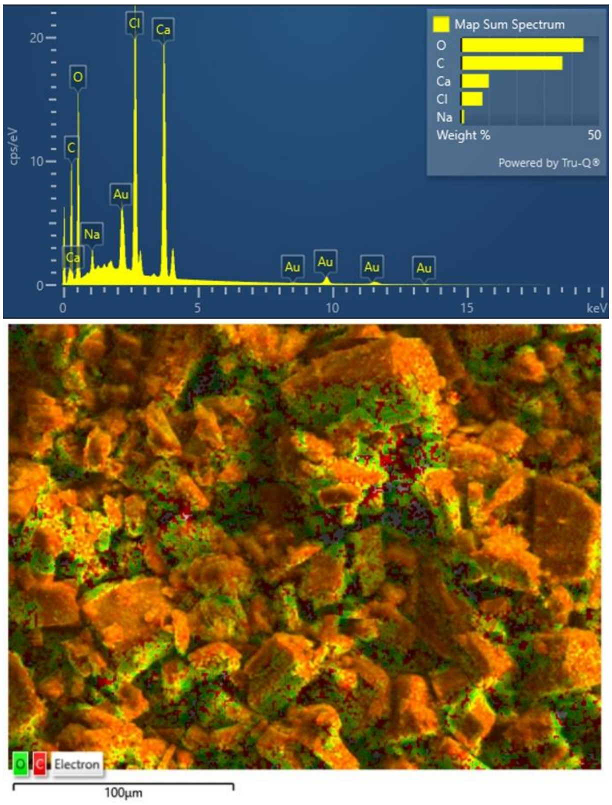
Map Sum Spectrum
O
C
Ca
Cl
Na
Weight %
50
Powered by Tru-Q®
cps/eV
keV
O C Electron
100µm

(c)

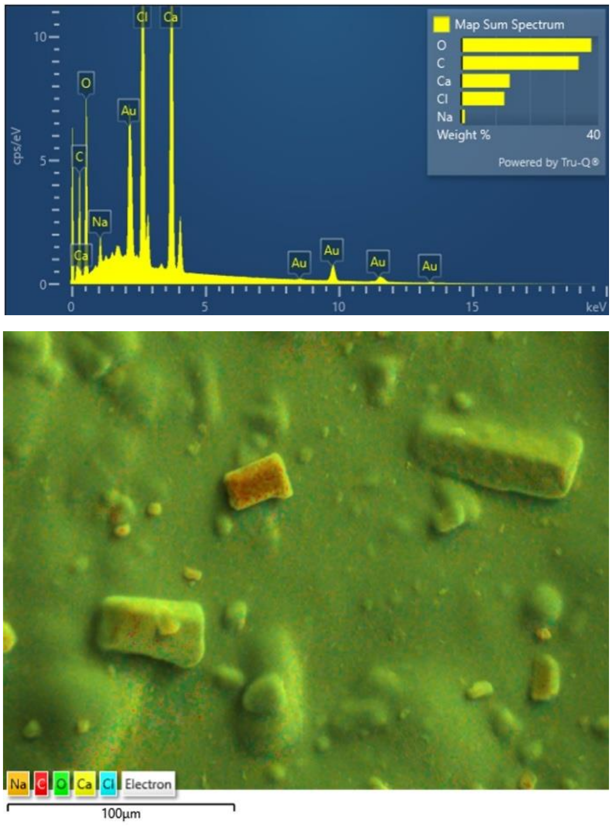


(d)

**Figure S2** – Calibration curve for *Curcuma longa* L. (CR) quantification obtained by UV-Vis spectroscopy at λ = 430 nm. The plot displays absorbance as a function of CR concentration (mg/L), used to calculate the amount of active compound released from the hydrogels. The linear regression equation and correlation coefficient ($R^2$) are presented in the graph.

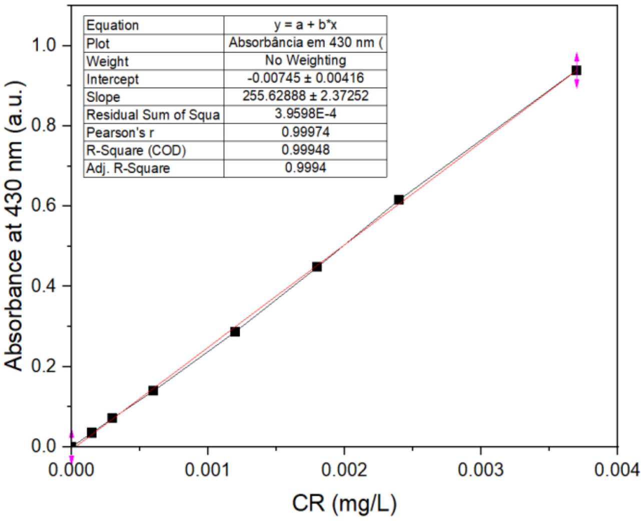

**Figure S3** Linear regression plots of *Curcuma longa* L. release kinetics from calcium alginate hydrogels (0.5% and 1.0% w/v) fitted to Zero-order, First-order, Higuchi, and Korsmeyer–Peppas models, respective.

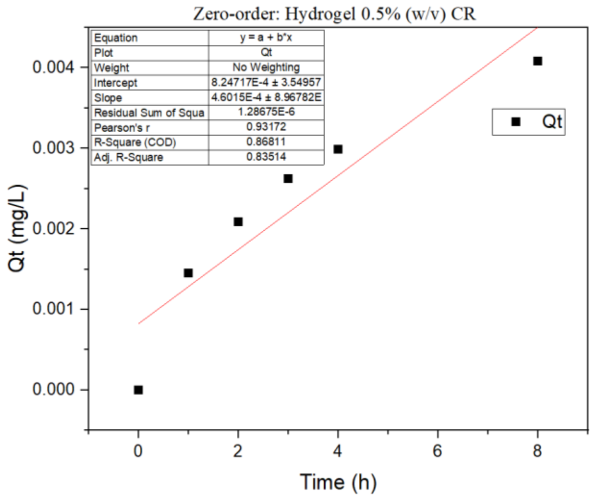


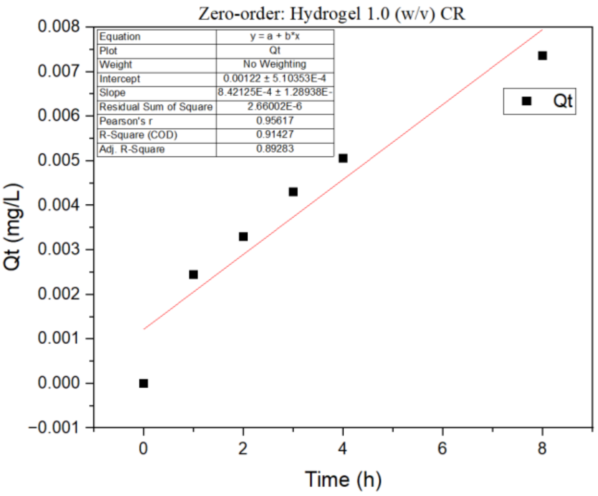


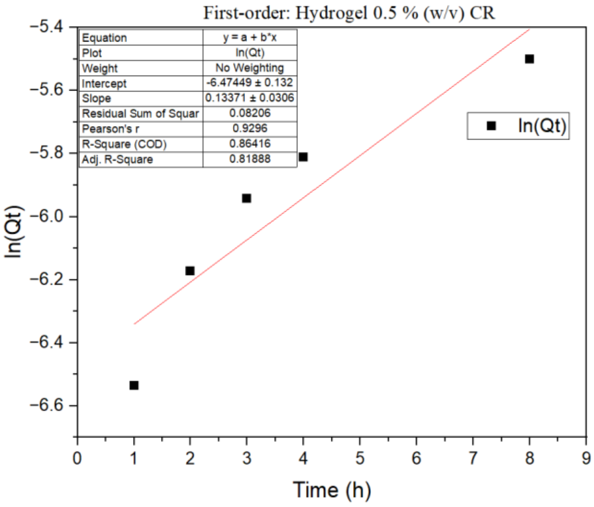

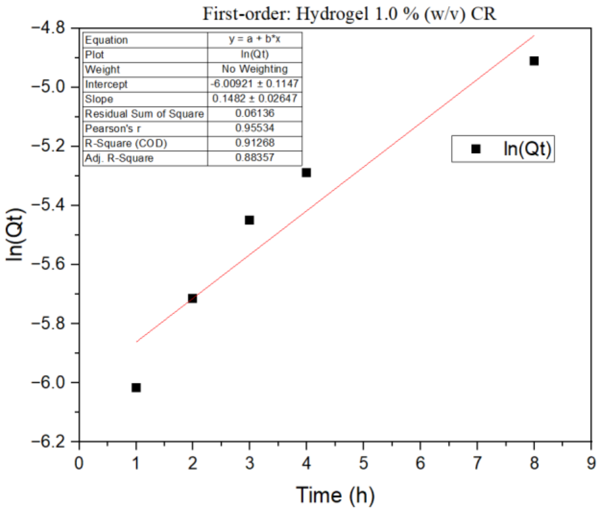
First-order: Hydrogel 1.0 % (w/v) CR
Equation | y = a + b*x
Plot | ln(Qt)
Weight | No Weighting
Intercept | -6.00921 ± 0.1147
Slope | 0.1482 ± 0.02647
Residual Sum of Square | 0.06136
Pearson's r | 0.95534
R-Square (COD) | 0.91268
Adj. R-Square | 0.88357
ln(Qt)
Time (h)

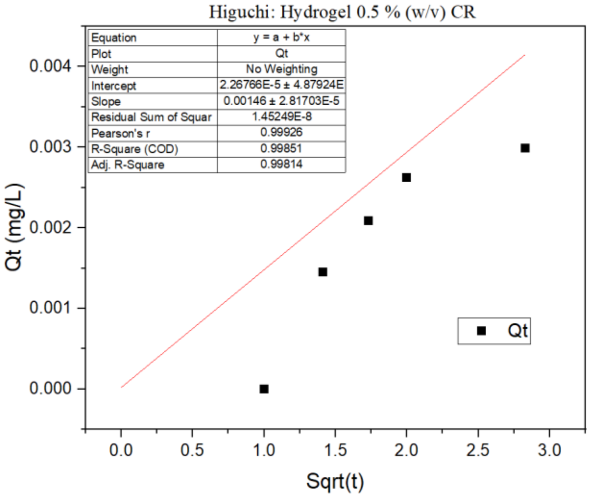
Higuchi: Hydrogel 0.5 % (w/v) CR
Equation | y = a + b*x
Plot | Qt
Weight | No Weighting
Intercept | 2.26766E-5 ± 4.87924E
Slope | 0.00146 ± 2.81703E-5
Residual Sum of Squar | 1.45249E-8
Pearson's r | 0.99926
R-Square (COD) | 0.99851
Adj. R-Square | 0.99814
Qt
Qt (mg/L)
Sqrt(t)

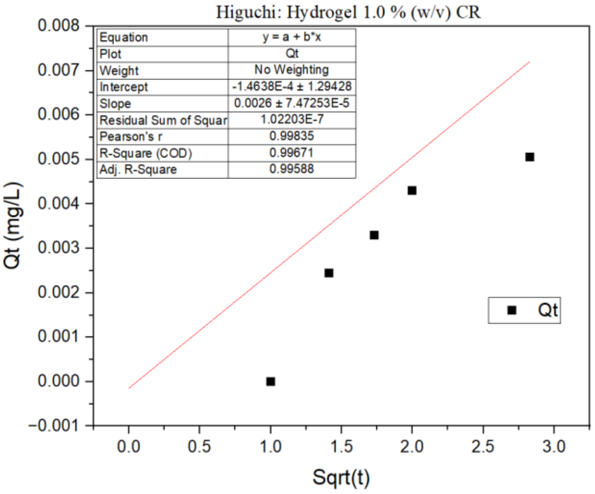
Higuchi: Hydrogel 1.0 % (w/v) CR
Equation | y = a + b*x
Plot | Qt
Weight | No Weighting
Intercept | -1.4638E-4 ± 1.29428
Slope | 0.0026 ± 7.47253E-5
Residual Sum of Squar | 1.02203E-7
Pearson's r | 0.99835
R-Square (COD) | 0.99671
Adj. R-Square | 0.99588
Qt
Qt (mg/L)
Sqrt(t)

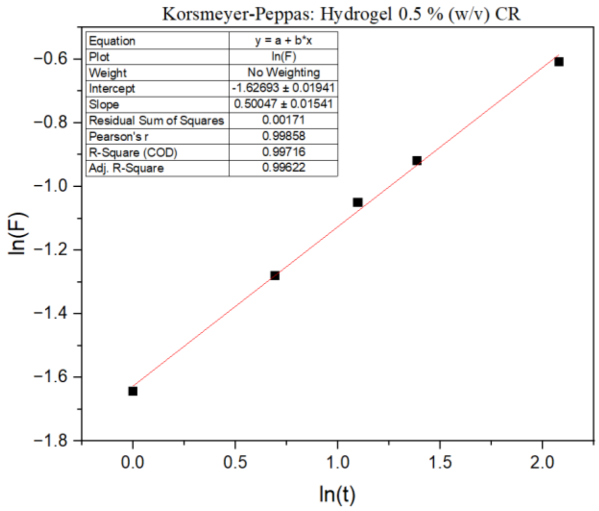
Korsmeyer-Peppas: Hydrogel 0.5 % (w/v) CR
Equation | y = a + b*x
Plot | ln(F)
Weight | No Weighting
Intercept | -1.62693 ± 0.01941
Slope | 0.50047 ± 0.01541
Residual Sum of Squares | 0.00171
Pearson's r | 0.99858
R-Square (COD) | 0.99716
Adj. R-Square | 0.99622
ln(F)
ln(t)

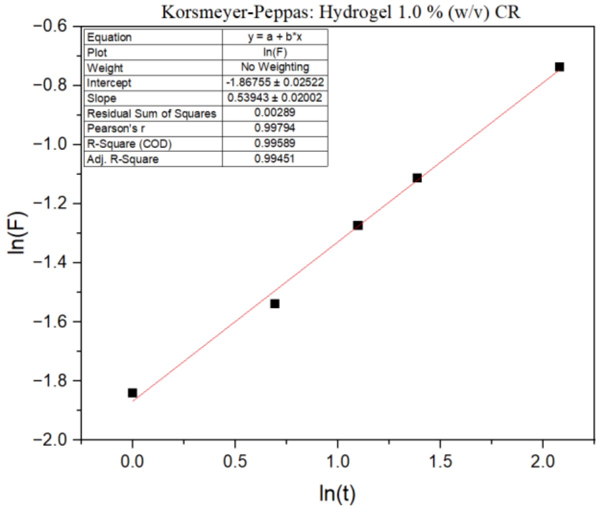
Korsmeyer-Peppas: Hydrogel 1.0 % (w/v) CR
Equation | y = a + b*x
Plot | ln(F)
Weight | No Weighting
Intercept | -1.86755 ± 0.02522
Slope | 0.53943 ± 0.02002
Residual Sum of Squares | 0.00289
Pearson's r | 0.99794
R-Square (COD) | 0.99589
Adj. R-Square | 0.99451
ln(F)
ln(t)
